# Cholesterol accelerates the binding of Alzheimer's β-amyloid peptide to ganglioside GM1 through a universal hydrogen-bond-dependent sterol tuning of glycolipid conformation

**DOI:** 10.3389/fphys.2013.00120

**Published:** 2013-06-10

**Authors:** Jacques Fantini, Nouara Yahi, Nicolas Garmy

**Affiliations:** EA-4674, Interactions Moléculaires et Systèmes Membranaires, Aix-Marseille UniversitéMarseille, France

**Keywords:** Alzheimer, cholesterol, ganglioside, GM1, lipid raft, lipid–lipid interaction, Langmuir monolayer, molecular modeling

## Abstract

Age-related alterations of membrane lipids in brain cell membranes together with high blood cholesterol are considered as major risk factors for Alzheimer's disease. Yet the molecular mechanisms by which these factors increase Alzheimer's risk are mostly unknown. In lipid raft domains of the plasma membrane, neurotoxic Alzheimer's beta-amyloid (Abeta) peptides interact with both cholesterol and ganglioside GM1. Recent data also suggested that cholesterol could stimulate the binding of Abeta to GM1 through conformational modulation of the ganglioside headgroup. Here we used a combination of physicochemical and molecular modeling approaches to decipher the mechanisms of cholesterol-assisted binding of Abeta to GM1. With the aim of decoupling the effect of cholesterol on GM1 from direct Abeta-cholesterol interactions, we designed a minimal peptide (Abeta5-16) containing the GM1-binding domain but lacking the amino acid residues involved in cholesterol recognition. Using the Langmuir technique, we showed that cholesterol (but not phosphatidylcholine or sphingomyelin) significantly accelerates the interaction of Abeta5-16 with GM1. Molecular dynamics simulations suggested that Abeta5-16 interacts with a cholesterol-stabilized dimer of GM1. The main structural effect of cholesterol is to establish a hydrogen-bond between its own OH group and the glycosidic-bond linking ceramide to the glycone part of GM1, thereby inducing a tilt in the glycolipid headgroup. This fine conformational tuning stabilizes the active conformation of the GM1 dimer whose headgroups, oriented in two opposite directions, form a chalice-shaped receptacle for Abeta. These data give new mechanistic insights into the stimulatory effect of cholesterol on Abeta/GM1 interactions. They also support the emerging concept that cholesterol is a universal modulator of protein-glycolipid interactions in the broader context of membrane recognition processes.

## Introduction

Age and high blood cholesterol are among the major non-genetic risk factors for Alzheimer's disease (Pappolla et al., 2003; Mayeux and Stern, 2012). We still do not know exactly why these factors increase Alzheimer's risk. However, a growing body of evidence suggests that the plasma membrane of neural cells plays a key role in the pathophysiology of the disease (Lukiw, 2013). Analyses of the lipid content of brain cell membranes during aging have revealed an increase in several types of lipids, including cholesterol and sphingolipids (Shinitzky, 1987). These lipids are concentrated in plasma membrane microdomains referred to as lipid rafts (Fantini et al., 2002). By modulating the lipid content of lipid rafts, age and high cholesterol could synergetically affect the organization and the physico-chemical properties of these domains, providing a favorable environment for the oligomerization and/or aggregation of Alzheimer's β-amyloid peptides (Di Paolo and Kim, 2012).

The proteolytic cleavage of the Alzheimer's protein precursor APP is a cholesterol-dependent process that occurs in lipid rafts (Ehehalt et al., 2003). Alzheimer's β-amyloid peptides Aβ1-40 and Aβ1-42 have a high affinity for these microdomains (Fantini and Yahi, 2010). Indeed, β-amyloid peptides interact with GM1, a ganglioside abundantly expressed in neural cell membranes and concentrated in lipid rafts (Ariga et al., 2011). A large body of data has conclusively demonstrated that GM1 plays a central role in the generation of toxic Aβ fibrils (Choo-Smith et al., 1997; Kakio et al., 2003; Hayashi et al., 2004; Wakabayashi et al., 2005; Chi et al., 2007; Matsuzaki et al., 2007, 2010; Okada et al., 2007; Yanagisawa, 2011; Matsubara et al., 2013). Interestingly, the interaction of Aβ with GM1 is cholesterol-dependent (Kakio et al., 2001; Okada et al., 2008; Yahi et al., 2010). Specifically, increasing the cholesterol content of lipid vesicles has been shown to facilitate the binding of Aβ to the membrane by altering the binding capacity, but not the binding affinity (Kakio et al., 2001).

There are two possible mechanisms by which cholesterol could improve the binding of Aβ peptides to GM1/cholesterol membranes. On one hand, Aβ could directly interact with cholesterol. On the other hand, cholesterol could indirectly affect Aβ binding to GM1 through a modulation of ganglioside conformation. As a matter of fact, Aβ contains a high affinity cholesterol-binding domain (segment 22–35) allowing a functional interaction of the peptide with membrane cholesterol (Di Scala et al., 2013). Moreover, direct binding of GM1 to Aβ has been evidenced through different experimental approaches including NMR (Williamson et al., 2006; Utsumi et al., 2009; Yagi-Utsumi et al., 2010), fluorescence titration (Ikeda and Matsuzaki, 2008), atomic force microscopy (Matsubara et al., 2013), and Langmuir monolayers (Thakur et al., 2009; Fantini and Yahi, 2011). The GM1-binding domain of Aβ has been delineated to a linear segment encompassing amino acid residues 5–16 (Fantini and Yahi, 2011). Because the binding sites for GM1 and cholesterol do not overlap, it can be assumed that Aβ1-40 and Aβ1-42 can bind to both lipids in a lipid raft domain. This particular situation renders difficult the experimental study of the second theoretical mechanism of cholesterol-stimulated Aβ binding to GM1, i.e., a conformational effect of cholesterol on GM1.

In the present study, we circumvent this difficulty by analyzing the effect of cholesterol on the interaction between GM1 and Aβ5-16, a functional GM1-binding peptide (Fantini and Yahi, 2011) that does not contain the cholesterol-binding domain of Aβ. We showed that cholesterol accelerates the interaction between Aβ5-16 and GM1 through a hydrogen-bond-driven conformational effect involving the glycone part of GM1. These data shed some light on the molecular mechanisms by which cholesterol and GM1 cooperate to boost the association of Aβ with lipid raft domains. From a broader perspective, this study is in line with the emerging concept that cholesterol functions as an intramembrane switch that controls ligand binding to GSL receptors (Fantini and Yahi, 2010; Yahi et al., 2010; Coskun and Simons, 2011; Lingwood, 2011; Lingwood et al., 2011).

## Materials and methods

### Materials

Kerasin (galactosylceramide, GalCer), GM1, and GM3 were obtained from Matreya (Pleasant Gap, PA). Lactosylceramide (LacCer) was purchased from Avanti Polar Lipids (Alabaster, AL). Cholesterol, sphingomyelin (SM), palmitoyl-oleoyl-phosphatidylcholine (POPC), and the Aβ1-40 peptide were from Sigma (Saint-Quentin Fallavier, France). The Aβ5-16 peptide was from Schafer (Denmark). All lipids were dissolved at a concentration of 1 mg.mL^−1^ in hexane:chloroform:ethanol (11:5:4, vol:vol:vol). Ultrapure water (pH 7.0, surface tension 72.8 mN.m^−1^, resistivity >18.2 MΩ.cm) was obtained from Biorad (Marnes-La-Coquette, France).

### Lipid monolayer assay

Peptide-lipid interactions were studied at 25°C with the Langmuir film balance technique (Thakur et al., 2009). The interaction of a peptide with a reconstituted membrane is an interfacial phenomenon which can be studied by surface pressure (π) measurements of lipid monolayers at the air–water interface. The underlying idea is that the insertion of the peptide in the lipid monolayer can be detected, at constant area, by an increase in the surface pressure. This increase in the surface pressure is caused by the insertion of the peptide between the polar heads of vicinal glycolipids in the monolayer, which is not counterbalanced by an increase of the area of the monolayer. This effect can be followed kinetically by real-time surface pressure measurements after injecting the peptide into the aqueous subphase underneath the lipid monolayer as described previously (Fantini et al., 2006; Yahi et al., 2010). Monomolecular films of the indicated lipid were spread on ultrapure water subphases totally devoid of any surfactant contaminant. To allow comparative studies, all monolayers were prepared at an initial surface pressure of 17–20 mN.m^−1^, which corresponds to a fully compressible film. After spreading of the film, 5 min was allowed for solvent evaporation. The Aβ5-16 peptide (fresh monomeric solution used at a final concentration of 10^−5^ M in ultrapure water) was then injected in the subphase (pH 7) with a 10-μl Hamilton syringe, and pressure increases produced were continuously recorded as a function of time. The data were analyzed with the FilmWareX program (Kibron Inc.). The accuracy of the system under our experimental conditions was ±0.25 mN.m^−1^ for surface pressure. The initial velocity (v_i_) of the insertion process is expressed as mN.m^−1^.min^−1^. The difference between the maximal (π_max_) and the initial (π_i_) surface pressure values allows to calculate the maximal surface pressure increase (Δπ_max_) induced by the peptide (expressed in mN.m^−1^). Mixed monolayers (Hammache et al., 2000) were prepared from stock solutions of lipid mixtures immediately before use.

### In silico studies

The starting structure of Aβ1-40 (Di Scala et al., 2013) and of Aβ5-16 (Fantini and Yahi, 2011) were derived from a NMR structure of Aβ1-40 in solution in a water–micelle environment (Coles et al., 1998), using the PDB entry 1BA4. Geometry optimization was first achieved using the unconstrained optimization rendered by the Polak–Ribière conjugate gradient algorithm. Molecular dynamics simulations were then performed for various periods of times ranging from 10 ps to 10 ns in vacuo with the Bio+ (CHARMM) force field (Singh et al., 2009) of the Hyperchem software suite (ChemCad, Obernay, France). The energy of interaction was determined with the Molegro Molecular Viewer (Thomsen and Christensen, 2006). Galactose-cholesterol and GM1-cholesterol models were obtained with the Hyperchem program as described previously (Yahi et al., 2010), by analogy with the GSL structures published by Pasher and Sundell (1977). Lipid-protein complexes were visualized with the PyMOL Molecular Graphics System, Version 1.2r3pre, Schrödinger, LLC.

## Results

### Cholesterol stimulates the interaction of Aβ1-40 with GSLs

**Figure 1:**
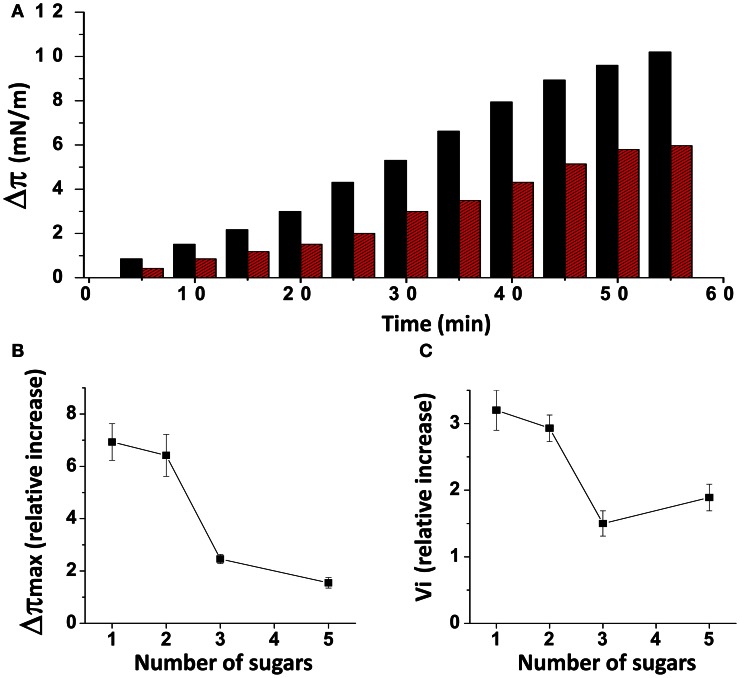
**Effects of cholesterol on GSL-Aβ1-40 interactions. (A)** Kinetics of Aβ1-40 insertion into a monolayer of GM1 in either the absence (red bars) or presence of cholesterol (black bars). The data show the evolution of the surface pressure following the injection of Aβ1-40 (1 μM) in the aqueous subphase underneath the monolayer. Each experiment was performed in triplicate and one representative curve is shown (S.D. <10%). **(B)** Maximal surface pressure increase (Δπ_max_) induced by Aβ1-40 on various GSL monolayers. The GSLs differed in the number of sugar units in their glycone part: 1 (GalCer), 2 (LacCer), 3 (GM3), and 5 (GM1). **(C)** Mean increase of the initial velocity (v_i_) induced by Aβ1-40 on these GSL monolayers. Data are expressed as the mean ± S.D. of three independent experiments.

When injected underneath a monolayer of pure ganglioside GM1, the Aβ1-40 peptide induced a gradual increase of the surface pressure (Figure 1A). When the experiment was performed with a mixed cholesterol/GM1 monolayer (1/1, mol/mol), the interaction with Aβ1-40 was significantly accelerated, and the maximal surface pressure increase (Δπ_max_) induced by the peptide reached a highest value (10 mN.m^−1^ with cholesterol, compared with 6 mN.m^−1^ with GM1 alone after 1 h of incubation). As previously reported (Yahi et al., 2010), this stimulatory effect of cholesterol was observed with some GSLs other than GM1, i.e., GalCer, LacCer, and GM3. Yet it is interesting to note that the intensity of the cholesterol effect was clearly dependent on the number of sugar units that constitute the glycone part of the GSLs. Indeed, both the amplitude (Δπ_max_) and the v_i_ of the phenomenon decreased as the number of sugars increased (Figures 1B,C). As a matter of fact, all these mixed cholesterol/GSL monolayers contain the same molar amount of cholesterol. Accordingly, one can assume that Aβ1-40 does not directly interact with cholesterol, and that the peptide binds to the glycone part of the GSLs through a cholesterol-dependent mechanism (Yahi et al., 2010). In the present study, we have designed a new Aβ-derived peptide probe to investigate further the mechanistic basis of GM1, cholesterol and Aβ interactions in a raft-mimicking environment.

### Aβ5-16 contains the GM1-binding domain of Aβ1-40 but does not interact with cholesterol

**Figure 2:**
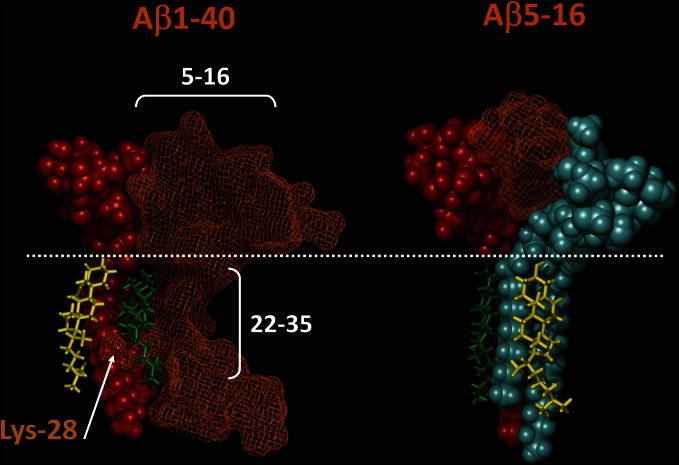
**Topology of lipid-binding domains in Aβ1-40 and Aβ5-16**. On the **left panel**, Aβ1-40 interacts with both GM1 (domain 5–16) and cholesterol (domain 22–35). In this complex, Aβ1-40 is in orange grid rendering, GM1 is rust-colored, and cholesterol is in green. Note the side chain of Lys-28 which wraps around the cholesterol molecule colored in green. Another cholesterol molecule (in yellow) interacts with the membrane-embedded part of GM1. The polar-apolar limit of the membrane is marked by a white dotted line. On the **right panel**, Aβ5-16 interacts with two GM1 molecules (rust and blue). Each of these GM1 molecules interacts with cholesterol. Note that these cholesterol molecules are embedded in the membrane (under the dotted line) and do not physically interact with Aβ5-16. These molecular models were obtained by molecular dynamics simulations as explained in Materials and Methods.

Our recent molecular modeling studies coupled with physico-chemical measurements of Aβ-lipid interactions suggested that the N-ter part of Aβ contains a glycolipid-binding domain (fragment 5–16) and the C-ter part a cholesterol-binding site (fragment 22–35). Yet we did not know whether the topology of Aβ in a membrane environment is consistent with dual recognition of GM1 and cholesterol. To assess whether the same Aβ1-40 peptide could interact with both GM1 and cholesterol, we performed a new series of molecular dynamics simulations. We used the individual structures of GM1-bound Aβ5-16 (Fantini and Yahi, 2011) and cholesterol-bound Aβ22-35 (Di Scala et al., 2013), and reintroduced them in the structure of a micellar, membrane-consistent topology of Aβ1-40 (Coles et al., 1998). The orientation of cholesterol in this trimolecular complex determined the mode of insertion of the Aβ peptide in the membrane. Specifically, cholesterol was embedded in the membrane and the sugar part of GM1 was protruding toward the extracellular milieu. With this geometry, the GM1-binding domain (Aβ5-16) was totally excluded from the membrane whereas the cholesterol-binding site (Aβ22-35) was totally immersed in the membrane (Figure 2, left panel). There was no physical contact between cholesterol and the 5–16 fragment of Aβ. Correspondingly, a model of the Aβ5-16 peptide bound to a GM1/cholesterol membrane (Figure 2, right panel) could be generated, with the following properties: (1) Aβ5-16 interacts with GM1, but not with cholesterol; (2) cholesterol interacts with GM1 in such a way that it can influence the conformation of the sugar headgroup. For these reasons, studying the interaction of Aβ5-16 with a GM1/cholesterol membrane has the unique advantage of decoupling the direct binding of Aβ to GM1 from indirect cholesterol-mediated effects on GM1 conformation.

### Cholesterol stimulates the interaction of Aβ5-16 with GM1

**Figure 3:**
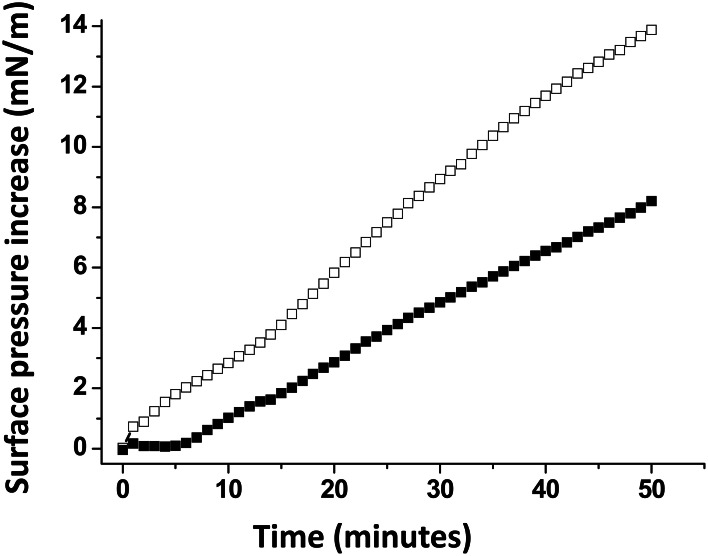
**Effect of cholesterol on the GM1-Aβ5-16 interaction**. The data show the evolution of the surface pressure following the injection of Aβ5-16 (10 μ M) in the aqueous subphase underneath a GM1 monolayer in either the absence (full squares) or presence of cholesterol (open squares). Each experiment was performed in triplicate and one representative curve is shown (S.D. <10%).

A monolayer of pure ganglioside GM1 was prepared and probed with the Aβ5-16 peptide added in the aqueous subphase (Figure 3). Following a lag time of 5 min, the surface pressure started to gradually increase, reaching 8 mN.m^−1^ after 50 min of incubation.

**Figure 4:**
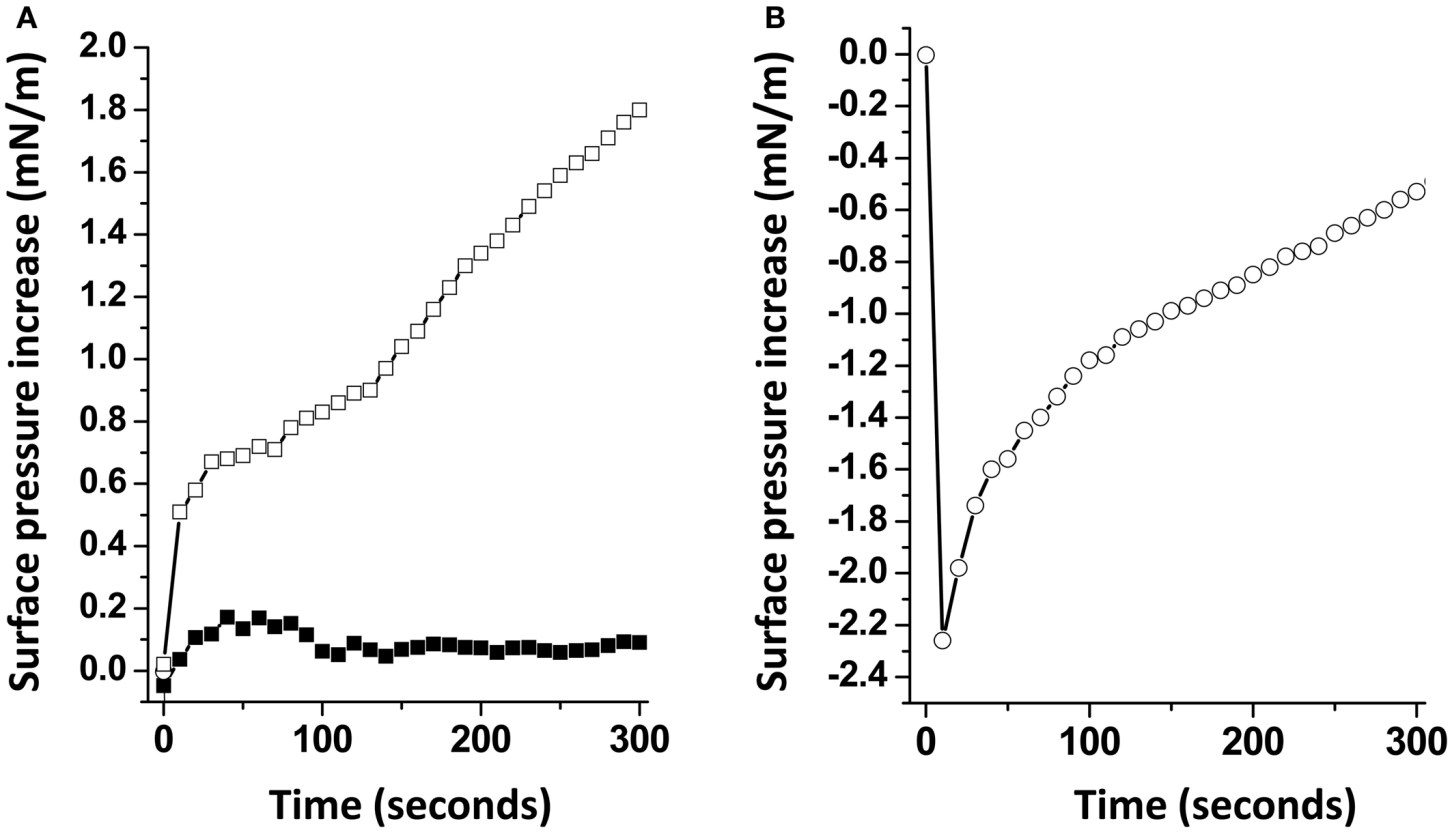
**Cholesterol accelerates the interaction of Aβ5-16 with GM1. (A)** Initial phase of interaction of Aβ-16 with pure GM1 (full squares) or mixed GM1-cholesterol monolayers (open squares). **(B)** Interaction of Aβ-16 with a cholesterol monolayer. Each experiment was performed in triplicate and one representative curve is shown (S.D. <10%).

When Aβ5-16 was injected underneath a mixed cholesterol/GM1 monolayer, the surface pressure increased immediately. After 50 min of incubation, the surface pressure increase was 14 mN.m^−1^. A detailed analysis of the first 5 min of incubation with the peptide confirmed the absence of lag phase in mixed cholesterol/GM1 monolayers (Figure 4A). In this case, the v_i_ of the interaction was estimated to 0.73 mN.m^−1^.min^−1^. For pure GM1 monolayers, (v_i_) was 0.06 mN.m^−1^.min^−1^. This corresponded to a strong cholesterol-evoked stimulation of v_i_ (× 12 times). For the sake of comparison, we studied the interaction of Aβ5-16 with a monolayer of pure cholesterol (Figure 4B). When the peptide was injected underneath this cholesterol monolayer, the surface pressure did not increase but first decreased and then gradually returned to null values. This indicated that the cholesterol-induced stimulation of Aβ5-16 binding to GM1 was not due to a direct interaction of the peptide with cholesterol, in full agreement with our modeling studies (Figure 2).

### The effect of cholesterol is specific

**Figure 5:**
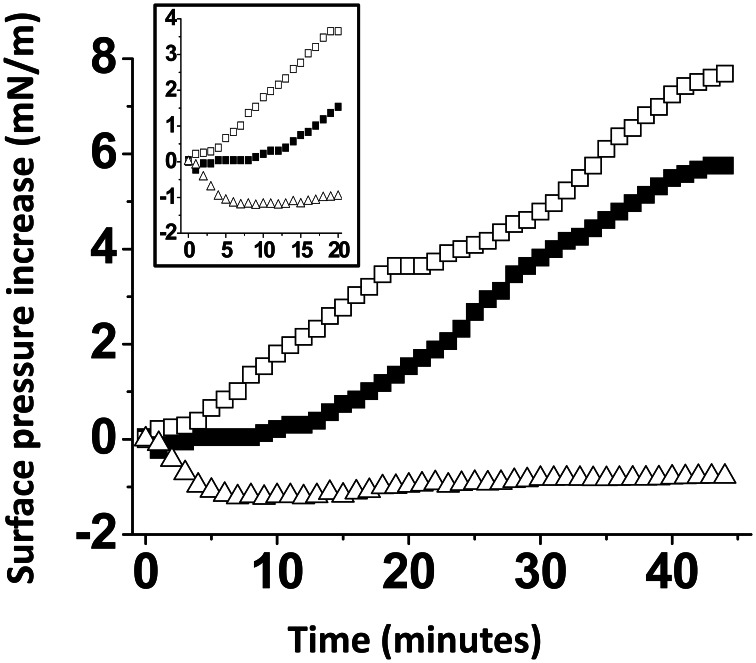
**The effect of cholesterol is specific**. Mixed GM1/SM (1:1, mol:mol), GM1/POPC (1:1, mol:mol), or GM1/POPC/cholesterol (2:1:1, mol:mol:mol) were prepared and probed with Aβ5-16 (10 μM) injected in the aqueous subphase. The data show the evolution of the surface pressure as a function of time following peptide injection underneath GM1/POPC (full squares), GM1/POPC/cholesterol (open squares), or GM1/SM (open triangles) monolayers. Each experiment was performed in triplicate and one representative curve is shown (S.D. <10%).

A possible interpretation of our data is that the presence of cholesterol within the GM1 monolayer could dilute the sugar headgroups of the ganglioside, resulting in an increase of accessibility of Aβ5-16 to GM1. To rule out this possibility, we prepared a series of GM1 monolayers mixed with various lipids (Figure 5). When the Aβ5-16 peptide was injected underneath a GM1/sphingomyelin monolayer (1:1, mol:mol), the surface pressure first decreased and then remained below the baseline during more than 40 min. Thus, not every membrane lipid could exert the stimulatory effect of cholesterol on the GM1/Aβ5-16 interaction, sphingomyelin (SM) being a significant counterexample. When GM1 was mixed with palmytoyl-oleoyl-phosphatidylcholine (POPC), there was a lag phase of 10 min before the peptide could induce any increase in the surface pressure (Figure 5). This indicated that dilution of GM1 in a phosphatidylcholine matrix was not sufficient to increase the v_i_ of the GM1/Aβ5-16 interaction (compare the kinetics of interaction of pure GM1 with mixed GM1/phosphatidylcholine monolayers in Figures 4, 5). Thus, phosphatidylcholine is another counterexample showing that the effect of cholesterol on the GM1/Aβ5-16 interaction is highly specific. Interestingly, when a molar fraction of phosphatidylcholine was replaced with cholesterol in a mixed GM1/POPC/cholesterol (2:1:1, mol:mol:mol) monolayer, the lag phase was no longer observed and the surface pressure increased immediately (although more slowly than with dual GM1/cholesterol monolayers) after the injection of the peptide (Figure 5, inset).

### Cholesterol constrains GSL conformation through a unique molecular mechanism

**Figure 6:**
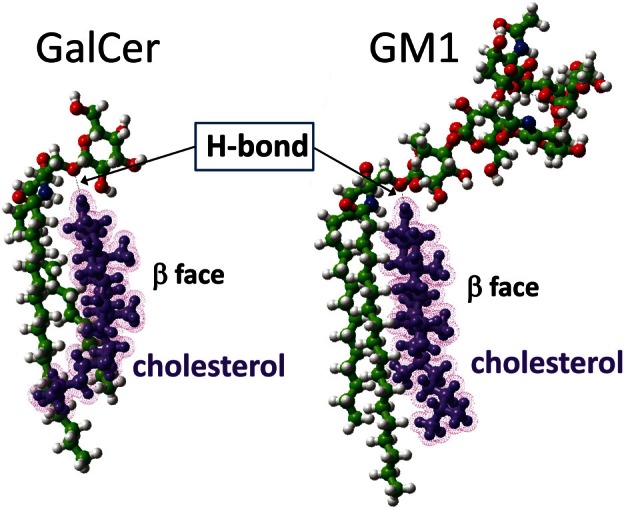
**Molecular dynamics simulations of cholesterol-GSL interactions**. In the cholesterol-GalCer complex **(left panel)**, the OH of cholesterol is a donor group that forms a hydrogen bond with the oxygen atom of the glycosidic linkage between galactose and ceramide. A similar [O–H…O] hydrogen bond is formed between cholesterol and GM1 **(right panel)**. In both cases, cholesterol constrains the GSL to adopt a typical shovel-like conformation. The other contacts between cholesterol and the GSL give rise to stabilizing van der Waals interactions between the apolar ceramide part of the GSL and the “smooth” α face of cholesterol. The “spiked” β face of cholesterol generally interacts with vicinal proteins in the membrane.

As recently reported by our group (Yahi et al., 2010), cholesterol induces a typical “shovel-like” conformation of kerasin (i.e., GalCer with a non-hydroxylated fatty acyl chain), whose unique galactosyl unit forms an angle of ca. 90° with the ceramide backbone (Figure 5). This conformation is stabilized by a hydrogen bond involving the OH group of cholesterol (donor H-bond group) and the oxygen atom of the glycosidic bond (acceptor H-bond group). In order to assess whether a similar hydrogen bond-driven mechanism could also account for a cholesterol-dependent effect on GM1 conformation, we performed a new series of molecular modeling simulations of cholesterol/GM1 interactions. As shown in Figure 6, the smooth α face of cholesterol spread along the ceramide backbone of GM1, leaving the OH group of cholesterol in front of the oxygen atom of the glycosidic bond. Thus, a hydrogen bond quite similar to the one of the GalCer/cholesterol complex could also stabilize the GM1/cholesterol complex. Correspondingly, this hydrogen bond kept the first sugar of GM1 (a glucosyl unit) parallel and flush to the membrane, inducing a tilt in the ganglioside structure.

### Cholesterol marginally affects the binding affinity of Aβ5-16 for GM1

**Figure 7:**
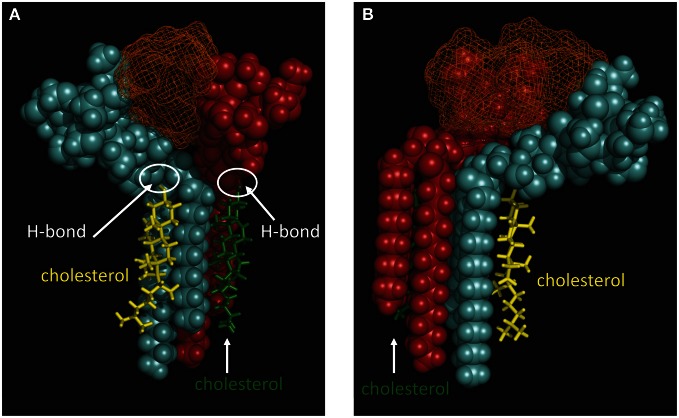
**Molecular modeling simulations of cholesterol-GM1-Aβ5-16 interactions**. Two distinct orientations of the models are shown: front view **(A)** and side view **(B)**. The shovel-like conformation of GM1 induced by cholesterol allows a specific topology of a couple of GM1 molecules interacting through their ceramide parts in the apolar phase of the membrane. Namely, the sugar parts of both GM1 molecules are oriented in two opposite directions, thereby delineating a chalice-shaped receptacle allowing an optimal interaction with Aβ5-16. The complex is stabilized by the H-bond between GM1 and cholesterol, and by a series of sugar-aromatic interactions between the glycone part of GM1 and specific amino acid side chains in Aβ5-16 (Table 1). These molecular models were obtained by molecular dynamics simulations as explained in Materials and Methods.

**Figure 8:**
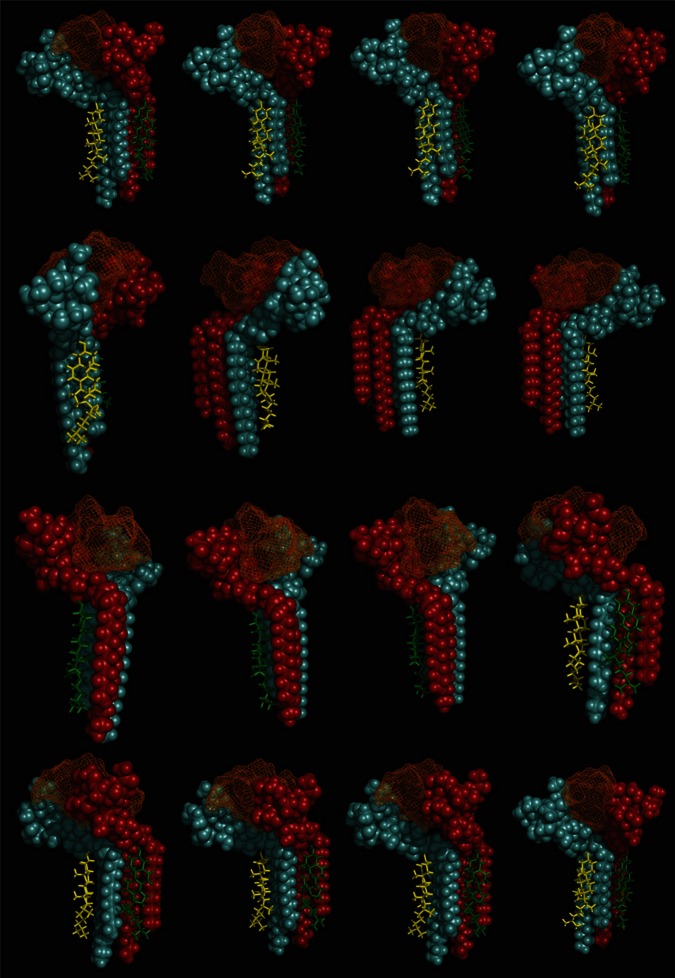
**Decomposed 360° rotation views of the cholesterol-GM1-Aβ5-16 complex**. Sixteen views resulting from regular 22.5° increments of the complex are shown.

Our modeling studies strongly suggested that the OH group of cholesterol restricts the conformation of the glycone part of various GSLs so that these GSLs adopt a typical L-shape structure (Figure 6). This tilted structure immediately suggests a specific orientation of the sugar headgroups in GM1 clusters. Nevertheless, we used different starting conditions to construct a dimer of the cholesterol/GM1 complex and we tested the thermodynamic stability of each complex by molecular dynamics simulations. The structure of the most stable dimer obtained by this way is shown in Figure 7. Both GM1 molecules interacted through their ceramide parts in the apolar phase of the membrane. Their respective sugar parts were rejected in two opposite directions, leaving a wide empty space that is fully compatible with the insertion of the whole Aβ5-16 peptide (Figure 7A). The typical shovel, L-like structure of GM1 is particularly well visible in the side view of the complex (Figure 7B). To get a better idea of the model, we have also shown a series of iterative views accounting for a whole 360° rotation of the complex (Figure 8). A detailed energetic analysis of the GM1/Aβ5-16 complex (without cholesterol) revealed that one GM1 molecule interacted chiefly with His-6, Glu-11, His-13, and Lys-16, whereas the other one interacted essentially with Arg-5, His-14, and Gln-15 (Table 1). Interestingly, the presence of cholesterol did not dramatically increase the energy of GM1/Aβ5-16 interaction. The main effect of GM1 was to improve the fit between GM1 and His-6 (from −6.8 to −22.5 kJ.mol^−1^). Otherwise, minor increases in the energy of interaction concerned Glu-11, Gln-15, and Lys-16. The total energy of interaction between Aβ5-16 and the GM1 dimer was estimated to −88.4 kJ.mol^−1^ without cholesterol, and 112.1 kJ.mol^−1^ with cholesterol (i.e., an increase of only × 1.3).

**Table 1:** **Energetics of interaction of Aβ5-16 with GM1: effect of cholesterol**.

**Amino acid residue in Aβ5-16**	**1st GM1 without cholesterol**	**1st GM1 with cholesterol**	**2nd GM1 without cholesterol**	**2nd GM1 with cholesterol**
Arg-5	−	−	−18.268	−19.292
His-6	−6.798	−22.475	−	−
Asp-7	−	−	−	−
Ser-8	−	−	−	−
Gly-9	−	−	−	−
Tyr-10	−	−	−	−
Glu-11	−13.545	−16.374	−	−
Val-12	−3.962	−3.179	−	−
His-13	−10.627	−9.988	−	−
His-14	−	−	−14.575	−15.585
Gln-15	−	−	−6.044	−8.721
Lys-16	−12.609	−13.499	−1.991	−3.024
Total	−47.541	−65.515	−40.878	−46.622

*The energy of interaction is determined after molecular docking of Aβ5-16 on GM1 in either the absence or presence of cholesterol (see Materials and Methods). In the model shown in Figure 7, the 1st GM1 molecule is colored in rust and the 2nd GM1 in blue*.

## Discussion

It is now widely admitted that cholesterol is a key regulator of membrane receptor function (Gimpl et al., 1997; Coskun and Simons, 2011; Lingwood, 2011). Cholesterol physically interacts with a broad range of membrane proteins (Fantini and Barrantes, 2009, 2013) through several types of cholesterol-binding domains including the consensus CRAC (Epand et al., 2010; Jafurulla et al., 2011; Picazo-Juárez et al., 2011) and CARC motifs (Baier et al., 2011), tilted peptides (Fantini et al., 2011), and three-dimensional sites (Hanson et al., 2008; Paila et al., 2009). Cholesterol also interacts with various sphingolipids such as sphingomyelin (Mattjus and Slotte, 1996), neutral GSLs (Slotte et al., 1993; Mahfoud et al., 2002), and gangliosides (Radhakrishnan et al., 2000; Taïeb et al., 2009). According to a new emerging concept, cholesterol has the unique capability to modulate GSL accessibility through direct conformational tuning of GSLs (Fantini and Yahi, 2010; Yahi et al., 2010; Coskun and Simons, 2011; Lingwood, 2011; Lingwood et al., 2011). By inducing a tilt in the GSL headgroup, cholesterol can either prevent or improve the accessibility of GSL receptors to extracellular ligands. Therefore, such GSLs may exist in two distinct states, their sugar headgroup being either parallel to the membrane (high cholesterol content) or protruding toward the extracellular space (low cholesterol content). This allows cholesterol to exert a binary control on GSL conformation and function. In this respect, this binary switch presents some analogy with the phosphorylation/dephosphorylation cycle of proteins, although in the case of cholesterol the regulation is non-covalent. A particularly convincing argument in favor of this concept comes from the study of sperm capacitation, a process associated with a loss of cholesterol (Cross, 1998). As elegantly shown by Lingwood et al. (2011), the reduction in membrane cholesterol levels in sperm cells evoked the unmasking of cryptic GSL receptor. Similarly, verotoxin binding to globotriaosylceramide (Gb_3_) in erythrocyte membrane was detected only after methyl-β-cyclodextrin removal of cholesterol from erythrocytes plasma membranes (Lingwood et al., 2011). The sudden exposure of the sugar headgroup of the GSL—that was previously maintained in a parallel orientation with respect to the membrane—explains why cholesterol depletion improves ligand binding.

In the case of Alzheimer's disease, the situation is completely different from that. The increase in cholesterol stimulates Aβ binding to GM1 and subsequently promotes β-amyloid fibrillation (Kakio et al., 2001), and this is consistent with the epidemiological link between high cholesterol and Alzheimer's disease. In a broader context, these data support the idea that cholesterol can exert opposite effects on GSL accessibility and/or function: (1) in some cases, ligand binding to a GSL receptor can be chronically inhibited by membrane cholesterol, and only a significant depletion of the sterol renders the GSL accessible to exogenous ligands (Lingwood et al., 2011); (2) in other cases, cholesterol improves GSL recognition by external ligands so that any increase in membrane cholesterol content improves the binding capacity of GSL-containing membranes (Kakio et al., 2001). Yet, these contrasting effects of cholesterol are due to a common molecular mechanism, i.e., the induction of a tilt in the GSL headgroup (Yahi et al., 2010; Lingwood et al., 2011).

A major outcome of the present study is that we could demonstrate the indirect effect of cholesterol on the GM1/Aβ interaction by using a truncated Aβ peptide (Aβ5-16) that recognizes GM1 but not cholesterol. Our physico-chemical data showed that the binding of Aβ5-16 to GM1 monolayer is dramatically accelerated (× 12) in presence of cholesterol (Figures 3, 4). This effect is highly specific since it was not observed when GM1 was mixed with either sphingomyelin or phosphatidylcholine (Figure 5). Thus we could totally rule out a non-specific “dilution” effect triggered by any lipid surrounding GM1 in the membrane. Accordingly, the effect of cholesterol could not be trivially interpreted as the result of lipid-mediated spacing of the sugar headgroups of ganglioside molecules. Indeed, replacing a molar fraction of phosphatidylcholine with cholesterol in a mixed GM1/phosphatidylcholine monolayer increased the v_i_ of the GM1/Aβ5-16 reaction (Figure 5). This further demonstrated that it is actually cholesterol, and not the presence of other “diluting” lipid molecules, that activates the binding of Aβ5-16 to GM1. In agreement with this notion, molecular dynamics simulations suggested that cholesterol helped GM1 to acquire a confirmation that favored the functional dimerization of GM1 gangliosides (Figure 7). With their respective sugar headgroups rejected in two opposite direction, the GM1 dimer formed a kind of chalice-shaped receptacle for Aβ5-16. Correspondingly, the interaction of Aβ5-16 with a mixed cholesterol/GM1 monolayer occurred with no lag phase (Figure 4A). It is likely that in absence of cholesterol, this typical conformation of GM1 molecules cannot occur spontaneously, but only after the mutual adaptation of GM1 and Aβ5-16 structures through an induced-fit mechanism. The latency observed before we could detect any interaction between Aβ5-16 and GM1 (Figure 4A) strongly supports this view. Moreover, estimations of the energy of interaction in GM1/Aβ5-16 in absence or presence of cholesterol (Table 1) suggest that cholesterol does not significantly increase the affinity of Aβ5-16 for GM1, in line with the notion that the effect of cholesterol is chiefly a kinetic one. This is supported by both physico-chemical and in silico data. By studying the interaction of various mutant peptides with GM1 monolayers, we identified the following amino acid residues of Aβ5-16 as critical for binding to GM1: Arg-5, His-13, His-14, and Lys-16, which act as primary binding sites for GM1 gangliosides (Fantini and Yahi, 2011). Cholesterol marginally affected the energy of interaction of these residues (Table 1). Instead, cholesterol reinforced the interaction with auxiliary residues such as His-6 and Gln-15, which act as accessory binding sites. Again, these data strongly suggest that cholesterol accelerates the interaction of Aβ with GM1 but does not dramatically increase the affinity of the peptide for its ganglioside receptors. By inducing a specific conformation of GM1, cholesterol facilitates the recruitment of these gangliosides into a functional dimeric unit able to bind the Aβ peptide without further conformational adjustment.

This conformational effect of cholesterol on GSLs is mostly due to the establishment of a hydrogen bond between the OH group of cholesterol (donor group) and the oxygen atom of the glycosidic bond linking the glycone part of the GSL to ceramide (acceptor group). Alternatively, the acceptor group for this hydrogen bond could be the OH in C2 of the first sugar (this OH group is oriented toward the plasma membrane and is located at hydrogen-bond-compatible distance from the OH of cholesterol). Such intermolecular hydrogen bonding interactions between two different lipids with suitable donor and acceptor groups (e.g., cholesterol and sphingolipids) have been previously characterized (Boggs, 1987; Nyholm et al., 1990). However, the involvement of the glycosidic bond (or alternatively the C_2_–OH of the first sugar) in these lipid–lipid hydrogen-bond-driven interactions is highly significant. Because it concerns the first glycosidic bond of the headgroup, the effect of cholesterol is particularly important for monohexosylceramides such as kerasin (Figure 1). Then the effect of cholesterol gradually decreases as the number of sugars increases. Nevertheless, it remains significant enough for GM1 (which contains five sugar units in the headgroup), so that it can be readily measured by the reasonably sensitive Langmuir monolayer assay (Figures 3, 4).

In conclusion, our data give new mechanistic insights into the stimulatory effect of cholesterol on Abeta/GM1 interactions. By increasing the local concentration of Aβ in lipid raft microdomains, cholesterol could either stimulate amyloid fibrillation (Yip et al., 2001; Matsuzaki et al., 2010) or facilitate amyloid channel formation (Micelli et al., 2004). In any case, this cholesterol/GM1-dependent polymerization of Aβ will elicit acute neurotoxic effects. On the opposite, lipid raft disruption has been shown to protect neurons against amyloid oligomer toxicity (Malchiodi-Albedi et al., 2010). Incidentally, our data also support the emerging concept that cholesterol is a universal modulator of protein-glycolipid interactions in the broader context of membrane recognition processes. This concept opens up new therapeutic strategies based on the design of synthetic GSL analogs in which the conformational effect of cholesterol is mimicked by a rigid chemical group such as adamantine (Mylvaganam and Lingwood, 1999; Mahfoud et al., 2002; Lund et al., 2006). It would be of high interest to consider such GSL/cholesterol-based therapies (Fantini, 2007) as an alternative approach for the treatment of Alzheimer's and other neurodegenerative diseases.

### Conflict of interest statement

The authors declare that the research was conducted in the absence of any commercial or financial relationships that could be construed as a potential conflict of interest.
